# An integrated lifestyle-, genetic- and metabolomics-based prediction model for mild cognitive aging in the HCHS/SOL

**DOI:** 10.1038/s44400-026-00120-9

**Published:** 2026-07-08

**Authors:** Annah B. Wyss, Yana Hrytsenko, Linfeng Hu, Iris J. Broce, Robert C. Kaplan, Wassim Tarraf, Bing Yu, Eric Boerwinkle, Qibin Qi, Myriam Fornage, Charles DeCarli, Hector M. González, Tamar Sofer

**Affiliations:** 1 CardioVascular Institute, Beth Israel Deaconess Medical Center, Boston, MA USA; 2 Department of Medicine, Harvard Medical School, Boston, MA USA; 3 Stanley Center for Psychiatric Research, Broad Institute of MIT and Harvard, Cambridge, MA USA; 4 Department of Neurosciences, University of California San Diego, La Jolla, CA USA; 5 Department of Epidemiology & Population Health, Albert Einstein College of Medicine, Bronx, NY USA; 6 Division of Public Health Sciences, Fred Hutchinson Cancer Research Center, Seattle, WA USA; 7 Institute of Gerontology & Department of Healthcare Sciences, Wayne State University, Detroit, MI USA; 8 Department of Epidemiology, School of Public Health, Human Genetics Center, The University of Texas Health Science Center at Houston, Houston, TX USA; 9 Institute of Molecular Medicine, McGovern Medical School, The University of Texas Health Science Center at Houston, Houston, TX USA; 10 Department of Neurology, University of California Davis, Davis, CA USA; 11 Department of Biostatistics, Harvard T.H. Chan School of Public Health, Boston, MA USA

**Keywords:** Biomarkers, Computational biology and bioinformatics, Diseases, Risk factors

## Abstract

Prediction models for cognitive aging measures have largely evaluated demographic variables and *APOE* carrier status in populations of European ancestry. To comprehensively assess prediction models among Hispanic/Latinos, we considered 12 models (6 predictor sets and 2 methods) for global cognitive score change (GCSC) and mild cognitive impairment (MCI) in the Study of Latinos-Investigation of Neurocognitive Aging (SOL-INCA) (*N* = 5856). Based on the average mean squared error (MSE) for GCSC or average area under the curve (AUC) for MCI across 100 randomly split testing and training sets, performance was similar across models, but slightly better for the following models: the chronic conditions model and genetic model (mean MSEs = 0.2464) using gradient-boosted trees for GCSC prediction and the chronic conditions model (mean AUC = 62%) and metabolite model (mean AUC = 60%) using logistic regression for MCI prediction. Using the Shapley Additive Explanations (SHAP) method, age at baseline, time between exams, and sex were the most important predictors for GCSC, followed by diabetes and global ancestral proportions. Diabetes and the metabolite ribitol had the highest influence on prediction of MCI. Although prediction performance was not especially high and did not vary greatly across models, incorporating information on diabetes, ancestry and metabolites may help improve prediction of GCSC and MCI.

## Introduction

The burden of Alzheimer’s Disease and Related Dementias (ADRD) is increasing in the United States (U.S.). In 2020, an estimated 6.1 million Americans aged 65 and older were living with Alzheimer’s disease (AD)^1^. By 2060, this estimate is projected to more than double to 13.8 million individuals^1^. Among racial and ethnic subgroups in the U.S., Hispanics/Latinos are predicted to have the largest increase in ADRD primarily due to the expected increase in the proportion of aged Hispanic/Latino individuals in the population^1,2^. Thus, it is important to understand and predict cognitive aging outcomes in Hispanic/Latino adults in the U.S.

Previous prediction models of cognitive aging outcomes have generally focused on select demographic variables (age, sex, and education), lifestyle variables (BMI and alcohol intake) and genetic factors (*APOE*-$\epsilon$4 carrier status)^3^. Using data from the Alzheimer’s Disease Neuroimaging (ADNI) study, recent prediction models have further sought to integrate broader genetic data. While numerous variants appeared useful in predicting AD, the number of genetic predictors considered outpaced sample sizes^4^. Additionally, previous prediction models have primarily been developed in populations of European ancestry, so these models may not have the same performance in populations with different predictor distributions or with other ancestral backgrounds^3,4^. Therefore, evaluation of more comprehensive prediction models which integrate lifestyle factors, co-morbidities, and omics (e.g., summary measures of genetics such as polygenic risk scores, PRS, and selected metabolites) among populations of diverse ancestry, such as Hispanic/Latinos, is warranted.

In particular, developing prediction models for cognitive aging outcomes among Hispanics/Latinos in the U.S. need to consider that such populations represent heterogenous cultures related to different countries of origin manifesting in different lifestyles and exposures, as well as genetic diversity due to historical migration patterns in these origin countries leading to diverse admixture patterns^5–9^. For example, one of the strongest known risk factors for AD is the *APOE*-$\epsilon$4 gene allele, but this association has been often reported as weaker in Hispanics/Latinos compared to non-Hispanic Whites^10,11^, and is not associated with mild cognitive impairment (MCI) in the Hispanic Community Health Study/Study of Latinos (HCHS/SOL), a large cohort study of Hispanics/Latinos in the U.S^12^. Considering genetic factors more broadly, a PRS for AD which included variants in the *APOE* region and many other regions had a stronger association than *APOE*-$\epsilon$4 or *APOE*-$\epsilon$2 alleles alone with MCI in HCHS/SOL^13^. Further, lifestyle factors, such as diet^14–17^, sleep duration^18–21^ and physical activity^22–26^, and comorbidities, such as hypertension^27–30^ and type 2 diabetes^31–35^, have all been shown to have an impact on cognitive function but distributions of such risk factors may be heterogenous in Hispanic/Latino populations. Finally, research from HCHS/SOL, ARIC and the Boston Puerto Rican Health Study (BPRHS), has identified several metabolites, mostly carbohydrates such as glucose and ribitol, consistently cross-sectionally associated with cognitive function in Hispanics/Latinos as well as other populations^36,37^.

**Fig. 1:**
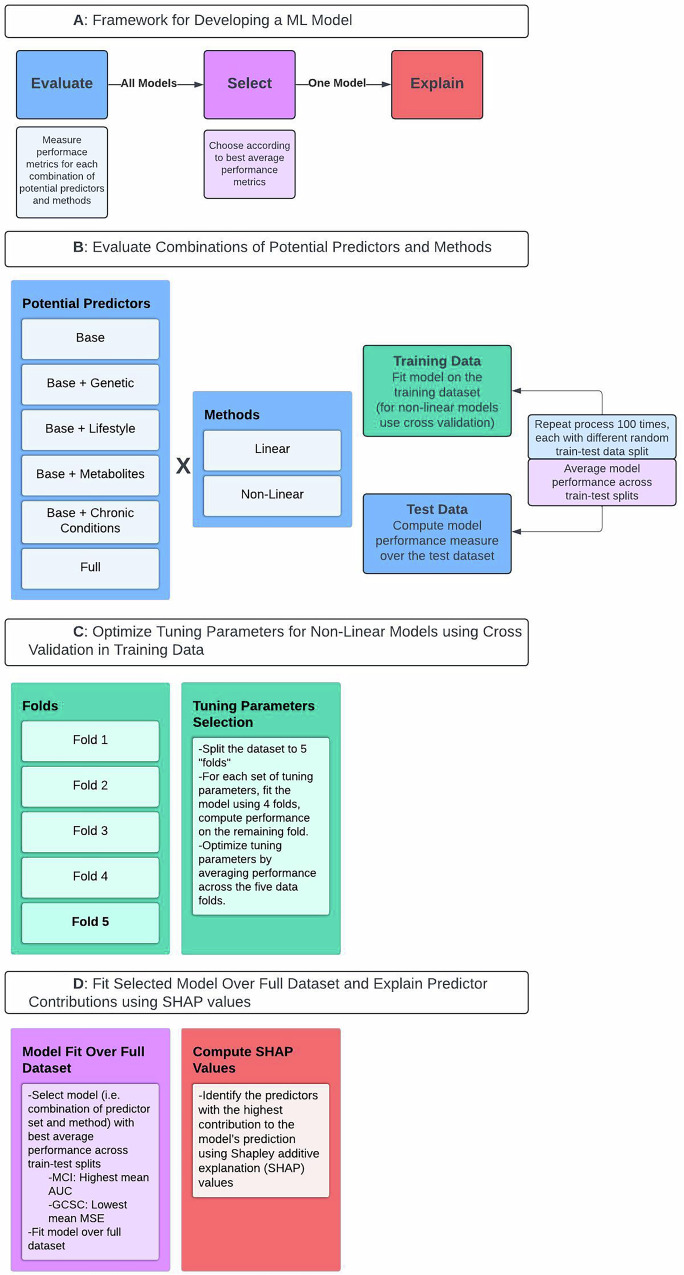
Framework for developing prediction models of GCSC and MCI. Panel **A** provides an overview on the three phases: Evaluate (detailed in Panel **B**), Select (detailed in Panel **D**) and Explain (detailed in Panel **D**). Panel **B** details the evaluation phase of prediction models based on combinations of 6 predictors sets and 2 methods. Average model performance was calculated across 100 train-test data splits. Panel **C** details tuning parameter optimization for non-linear models using 5-fold cross validation in the training dataset. Panel **D** details the selection phase in which the best prediction model (combination of preditor set and method) was selected based on lowest mean MSE for GCSC and highest mean AUC for MCI. Panel **D** also details the explanation phase in which features contributing the most to the selected prediction model were identified using SHAP values.

The present paper extends current prediction models and builds upon our previous findings from HCHS/SOL by comparing several prediction models for global cognitive score change (GCSC) and mild cognitive impairment (MCI) in this large cohort of Hispanics/Latinos in the U.S. GCSC shows changes in cognitive health over time, helping to track cognitive decline or improvement, making it useful for identifying individuals at-risk for more severe conditions such as MCI or ADRD. Likewise, MCI represents an important stage in cognitive decline and is a precursor to dementia. Although the conversion of such precursor conditions to dementia is complex and our models do not directly predict such disease progression, understanding GCSC and future MCI status is important for early intervention. Specifically, GCSC and MCI prediction models evaluated in the present paper were constructed based on six sets of predictors (including demographic, lifestyle, chronic condition, metabolomic, and genetic factors) and two methods (linear/logistic regression and gradient-boosted trees) (Fig. 1). Rather than performing individual variable selection, we compared models based on groups of risk factors to help tease out which types of data (e.g., questionnaire data on lifestyle variables or biologic data on genetics or metabolomics) are most important in predicting cognitive aging outcomes. Further, since the relationship between predictors and cognitive aging outcomes are complex and may differ by subgroups of the populations in a way that is difficult or not possible to pre-specify, using non-linear machine learning methods have the potential to efficiently use the predictors and improve outcome prediction^38^. In particular, methods utilizing gradient-boosted trees were recently shown to improve risk prediction in diverse human populations^39–42^. By evaluating several prediction sets and methods in HCHS/SOL we sought to better understand and ultimately improve prediction of cognitive measures in aged Hispanic/Latino populations.

## Results

### Participant characteristics

Global cognitive scores (GCS) were available for 9223 individuals at baseline and 5880 individuals at follow-up. Among those classified as having “poor” baseline GCS, 48.3% (715/1479) were missing follow-up GCS. Among those who were not classified as having “poor” baseline GCS, 33.9% (2628/7744) were missing follow-up GCS. After excluding participants missing GCSC or MCI, this analysis included 5,856 individuals. Among them, 558 (9.5%) met MCI criteria (Table 1). More females (*N* = 3773, 64.4%) than males were included in analyses, with the proportion of females similar between those with MCI (*N* = 375, 67.2%) and without MCI (*N* = 3398, 64.1%). The average age of individuals at baseline was around 54.9 years (56.7 years among those with MCI and 54.7 years among those without). The average time between the HCHS/SOL baseline visit and the Study of Latinos-Investigation of Neurocognitive Aging (SOL-INCA) follow-up visit was around 7 years. The average global ancestral proportions (GAPs) were 13% African, 57% European and 29% Indigenous American, which did not vary by MCI status. At baseline, 2701 (46.1%) individuals met diabetes criteria and 1554 (26.5%) pre-diabetes criteria. The proportion of individuals who had diabetes was noticeably higher among those with MCI (*N* = 226, 40.5%) compared to those without MCI (*N* = 1328, 25.1%). Over half the individuals in this analysis met hypertension criteria at baseline (*N* = 3373, 57.6%), again with a higher proportion of those with MCI having hypertension (*N* = 376, 67.4%) compared to those without MCI (*N* = 2997, 56.6%). Individuals reported an average of 7.8 h of sleep, 526.2 (MET-min/day) of physical activity, a BMI of 30.1 and a Mediterranean diet score of 3.7, which did not vary noticeably by MCI status other than physical activity which was slightly lower in individuals with MCI. The data was collected at 4 U.S.-based study centers including centers in Miami (*N* = 1633), San Diego (*N* = 1579), Chicago (*N* = 1480), and Bronx (*N* = 1164). Just under a quarter of individuals were carriers of 1 or 2 copies of APOE-4 (*N* = 1245, 21.3%), with a slightly higher proportion observed among those with MCI (23.8%) compared to those without MCI (21.0%). For APOE-2, under 9% of individuals were carriers (*N* = 497), with a slightly lower proportion among those with MCI (7.2%) compared to those without MCI (8.6%). Ribitol was not measured in batch 1, while the other 5 metabolites had missingness rates ranging from 0% to 9% in batch 1. In batch 2, missingness rates for the 6 metabolites ranged from 0% to 10%.

**Table 1:** Study characteristics at baseline of HCHS/SOL participants with and without MCI

Characteristics (mean, SD)	Overall (*N* = 5856)	Individuals with MCI (*N* = 558)	Individuals without MCI (*N* = 5298)
Global Cognitive Score Change	−0.04 (0.5)	−0.56 (0.5)	0.01 (0.5)
Years between visits	6.9 (1.2)	7.0 (1.2)	6.9 (1.2)
Age at baseline (years)	54.9 (7.1)	56.7 (7.9)	54.7 (7.0)
Global ancestry proportions (GAPs)			
African	0.13 (0.16)	0.13 (0.16)	0.13 (0.16)
European	0.57 (0.21)	0.57 (0.20)	0.57 (0.21)
Indigenous American	0.29 (0.23)	0.30 (0.22)	0.29 (0.23)
Sex (*N*, %)			
Female	3773 (64.4)	375 (67.2)	3398 (64.1)
Study center (*N*, %)			
Miami	1633 (27.9)	151 (27.1)	1482 (28.0)
San Diego	1579 (27.0)	169 (30.3)	1410 (26.6)
Chicago	1480 (25.3)	137 (24.6)	1343 (25.3)
Bronx area New York	1164 (19.9)	101 (18.1)	1063 (20.1)
Diabetes (*N*, %)			
Diabetes	1554 (26.5)	226 (40.5)	1328 (25.1)
Pre-diabetes	2701 (46.1)	214 (38.4)	2487 (46.9)
Hypertension (*N*, %)	3373 (57.6)	376 (67.4)	2997 (56.6)
Sleep duration (hours)	7.8 (1.4)	7.9 (1.5)	7.8 (1.3)
Physical activity (MET-min/day^a^)	526.2 (876.4)	484.5 (886.4)	530.7 (875.3)
BMI	30.1 (5.6)	30.7 (5.8)	30.0 (5.5)
Mediterranean Diet Score	3.7 (1.6)	3.6 (1.6)	3.7 (1.6)
APOE carrier (*N*, %)			
APOE 4	1245 (21.3)	133 (23.8)	1112 (21.0)
APOE 2	497 (8.5)	40 (7.2)	457 (8.6)
Metabolites (normalized concentrations)			
5’-Methylthioadenosine	0.06 (1.00)	0.11 (1.08)	0.05 (0.99)
Glucose	0.07 (0.99)	0.29 (1.08)	0.04 (0.98)
Mannose	0.04 (0.99)	0.32 (1.06)	0.02 (0.98)
Gamma-CEHC glucuronide	−0.01 (1.00)	0.05 (1.01)	-0.01 (1.01)
Mannitol/Sorbitol	0.06 (1.00)	0.27 (1.05)	0.04 (0.99)
Ribitol	0.01 (0.99)	0.28 (1.10)	−0.02 (0.97)

^a^MET-min/day = [(days/week × minutes/day of vigorous-intensity work × 8) + (days/week × minutes/day of moderate-intensity work × 4) + (days/week × minutes/day of transportation × 4) + (days/week × minutes/day of vigorous-intensity recreation × 8) + (days/week × minutes/day of moderate-intensity recreation × 4)]/7.

### GCSC model prediction

**Fig. 2:**
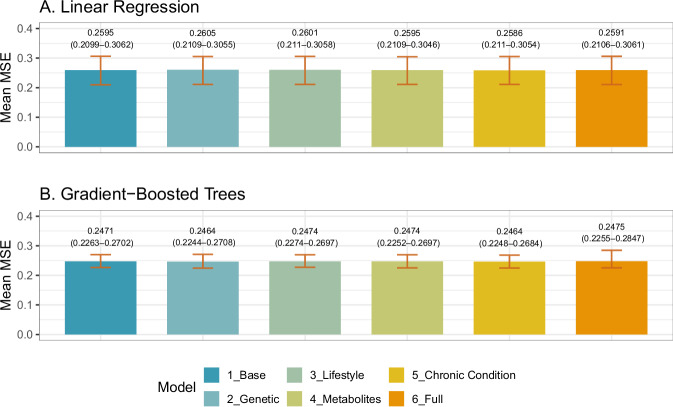
Model performance for predicting GCSC (mean squared error, MSE, and 95% confidence interval, CI) based on two methods and six predictor sets. Two methods include: linear regression (Panel **A**) and gradient-boosted trees (Panel **B**). Six predictor sets include: 1) Base (age, sex, BMI, time between exams), 2) Genetic (Base + APOE alleles, AD PRS, GAPs), 3) Lifestyle (Base + sleep duration, Mediterranean diet score, exercise), 4) Metabolites (Base + gamma-CEHC glucuronide, 5’-Methylthioadenosine, glucose, mannose, ribitol, and mannitol/sorbitol), 5) Chronic Conditions (Base + diabetes, hypertension), and 6) Full (base + genetic  + lifestyle + metabolites + chronic conditions).

For predicting GCSC, models using gradient-boosted trees had slightly lower mean squared errors (MSEs) than linear regression, suggesting non-linear methods may outperform linear methods in this setting. Averaging across 100 repeated training and test splits, the mean MSE across predictor sets ranged from 0.2464 to 0.2475 using gradient-boosted trees and from 0.2586 to 0.2605 using linear regression (Fig. 2, Supplementary Table 1). When comparing predictor sets using gradient-boosted trees, the chronic condition model (base + diabetes, hypertension; mean MSE = 0.2464, 95% CI = 0.2248–0.2684) and the genetic model (base + *APOE*-$\epsilon$2, *APOE*-$\epsilon$4, AD PRS, GAPs; MSE = 0.2464, 95% CI = 0.2244-0.2708) performed the best. However, other predictor sets, including the base model (mean MSE = 0.2471, 95% CI = 0.2263–0.2702), had similar mean MSE values. When comparing differences in MSE across models, the chronic condition (mean difference = −6.94 × 10^−4^, 95% CI = −9.12 × 10^−3^–5.72 × 10^−^^3^) and genetic (mean difference = −6.71 × 10^−4^, 95% CI = −8.55 × 10^−3^–9.96^−3^) models did not appear to differ substantially from the base model (Supplementary Table 2). Among the linear regression models, the chronic condition predictor set again had the lowest mean MSE (0.2586, 95% CI = 0.211–0.3054), though other models including the base model (mean MSE = 0.2595, 95% CI = 0.2099–0.3062) had similar mean MSE values. In sensitivity analyses, gradient-boosted trees models excluding missing data performed worse than both the primary gradient-boosted trees models which accommodate missing data and the linear regression models which exclude individuals missing data on predictors. For gradient-boosted trees models excluding missing data, the mean MSEs (95% CIs) ranged from 0.2615 (0.2102–0.3125) for the chronic condition model to 0.2656 (0.2203–0.3206) for the metabolite model (Supplementary Table 1).

Further, when we considered prediction models among individuals ≤55 years old and >55 years old separately, linear regression performed slightly better than gradient-boosted trees in both age groups (Supplementary Table 3). Among those ≤55 years old, the base model (mean MSE = 0.2601, 95% CI = 0.1443–0.2736) followed by the chronic condition model (mean MSE = 0.2067, 95% CI = 0.1447, 0.2728) using linear regression had slightly better performance than other models. The mean difference in MSE between these two models was 0.0007 (−0.0015–0.0042) (Supplementary Table 2). Among those >55 years old, the genetic model (mean MSE = 0.2532, 95% CI = 0.1935–0.3316) using linear regression had slightly better performance than other models, with a mean difference in AUC from the base model of −0.0045 (−0.0179–0.0140) (Supplementary Tables 2, 3). Comparing across age groups, all models performed slightly better among individuals ≤55 years old than individuals >55 years old. For example, the mean MSE (95% CI) for the chronic condition and genetic models were 0.2067 (0.1447–0.2728) and 0.2088 (0.1472–0.2747) among those ≤55 years old and 0.2576 (0.1932–0.3288) and 0.2532 (0.1935–0.3316) among those >55 years old (Supplementary Table 3). The mean difference in MSE (95% CI) across the age groups was 0.0508 (−0.0572–0.1375) for the chronic condition model and 0.0444 (−0.0598–0.1256) for genetic model using linear regression.

### MCI model prediction

**Fig. 3:**
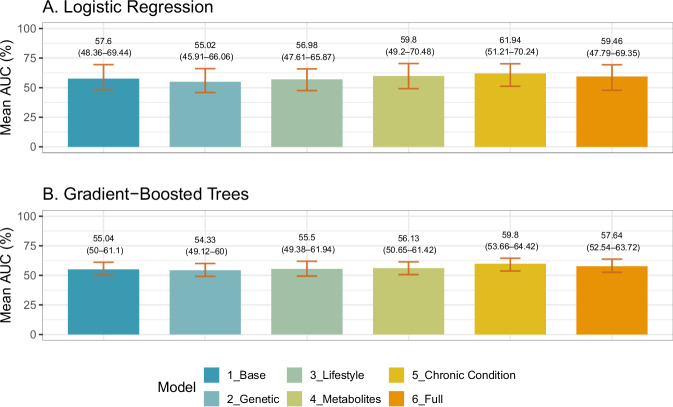
Model performance for predicting MCI (area under the curve, AUC and 95% confidence interval, CI) based on two methods and six predictor sets. Two methods include: logistic regression (Panel **A**) and gradient-boosted trees (Panel **B**). Six predictor sets include: 1) Base (age, sex, BMI, time between exams), 2) Genetic (Base + APOE alleles, AD PRS, GAPs), 3) Lifestyle (Base + sleep duration, Mediterranean diet score, exercise), 4) Metabolites (Base + gamma-CEHC glucuronide, 5’-Methylthioadenosine, glucose, mannose, ribitol, and mannitol/sorbitol), 5) Chronic Conditions (Base + diabetes, hypertension), and 6) Full (base + genetic  + lifestyle + metabolites+ chronic conditions).

For predicting the binary measure MCI, logistic regression appeared to outperform gradient-boosted trees based on higher area under the receiver operating characteristic (ROC) curve (AUC). The mean AUCs for logistic regression models ranged from 55.02% to 61.94% depending on the predictor set compared to 54.33% to 59.80% for gradient-boosted trees (Fig. 3, Supplementary Table 4). When comparing predictor sets using logistic regression, the chronic conditions model (base + diabetes, hypertension) had the best performance (mean AUC = 61.94%, 95% CI = 51.21–70.24) followed by the metabolite model (base + 6 metabolites, mean AUC = 59.80%, 95% CI = 49.20−70.48). However, other predictor sets, including the base model (mean AUC = 57.6%, 95% CI = 48.36−69.44), had similar mean AUC values. In secondary analyses, the metabolite model including diabetes also had similar performance (mean AUC = 61.80%). When comparing differences in AUC across models, the mean difference (95% CI) was 2.20% (−8.67–12.67) for the metabolite model and 4.35% (−4.49–13.24) for the chronic condition model compared to the base model (Supplementary Table 5). Unlike GCSC, the genetic model had the lowest performance for MCI (base + AD PRS, *APOE*-$\epsilon$4, *APOE*-$\epsilon$2, GAPs, mean AUC = 55.02%, 95% CI = 45.91–66.06, Supplemental Table 4). Similarly, when using gradient-boosted trees, the chronic condition (mean AUC = 59.80%, 95% CI = 53.66–64.42) and metabolite (mean AUC = 56.13%, 95% CI = 50.65–61.42) models were among models with the highest mean AUCs, and the genetic model had the lowest mean AUC (mean AUC = 54.33%, 95% CI = 4.12–60.00). In sensitivity analyses, gradient-boosted trees models excluding individuals with missing data had lower performance across most predictor sets, including for the chronic condition model (mean AUC = 52.71%, 95% CI = 48.09–65.18).

When considering mean F1, a measure of precision and recall, the chronic condition and metabolite models using logistic regression again displayed slightly better performance (mean F1 = 0.2170 and 0.2078 respectively) (Supplementary Table 4). However, when considering accuracy, gradient-boosted trees performed slightly better than logistic regression across models, with the base predictor set having the best performance (mean accuracy = 0.8968) followed closely by the chronic condition (mean accuracy = 0.8913) and metabolite (mean accuracy = 0.8903) predictors sets (Supplementary Table 4).

Finally, when we considered prediction models among individuals ≤55 years old and >55 years old separately, we again noted that the models did not drastically differ across predictors sets or methods, though the chronic condition and metabolite models using logistic regression appeared to have slightly better performance than other models (Supplementary Table 6). Within age groups, the mean difference in AUC (95% CI) between the chronic condition model and metabolite model compared to the base model using logistic regression was 4.81% (−2.88–11.77) and 6.24 (−11.53–21.89) among individuals ≤55 years old and 1.77% (−1.43–4.23) and 0.72% (−14.82–16.40) among individuals >55 years old (Supplementary Table 5). Interestingly, all models performed slightly better among individuals >55 years old than individuals ≤55 years old. For example, the mean AUC (95% CI) for the chronic condition and metabolite models using logistic regression were 60.32% (52.91–66.94) and 59.27% (45.01–70.64) among individuals >55 years old compared to 56.88% (50.92–64.28) and 58.32% (43.74–71.85) among individuals ≤55 years old (Supplementary Table 6). The mean difference in AUC (95% CI) across the age groups was 3.43% (−6.51–12.96) for the chronic condition model and 0.95% (−17.52–22.47) for metabolite model using logistic regression.

### Model selection and feature impact

**Fig. 4:**
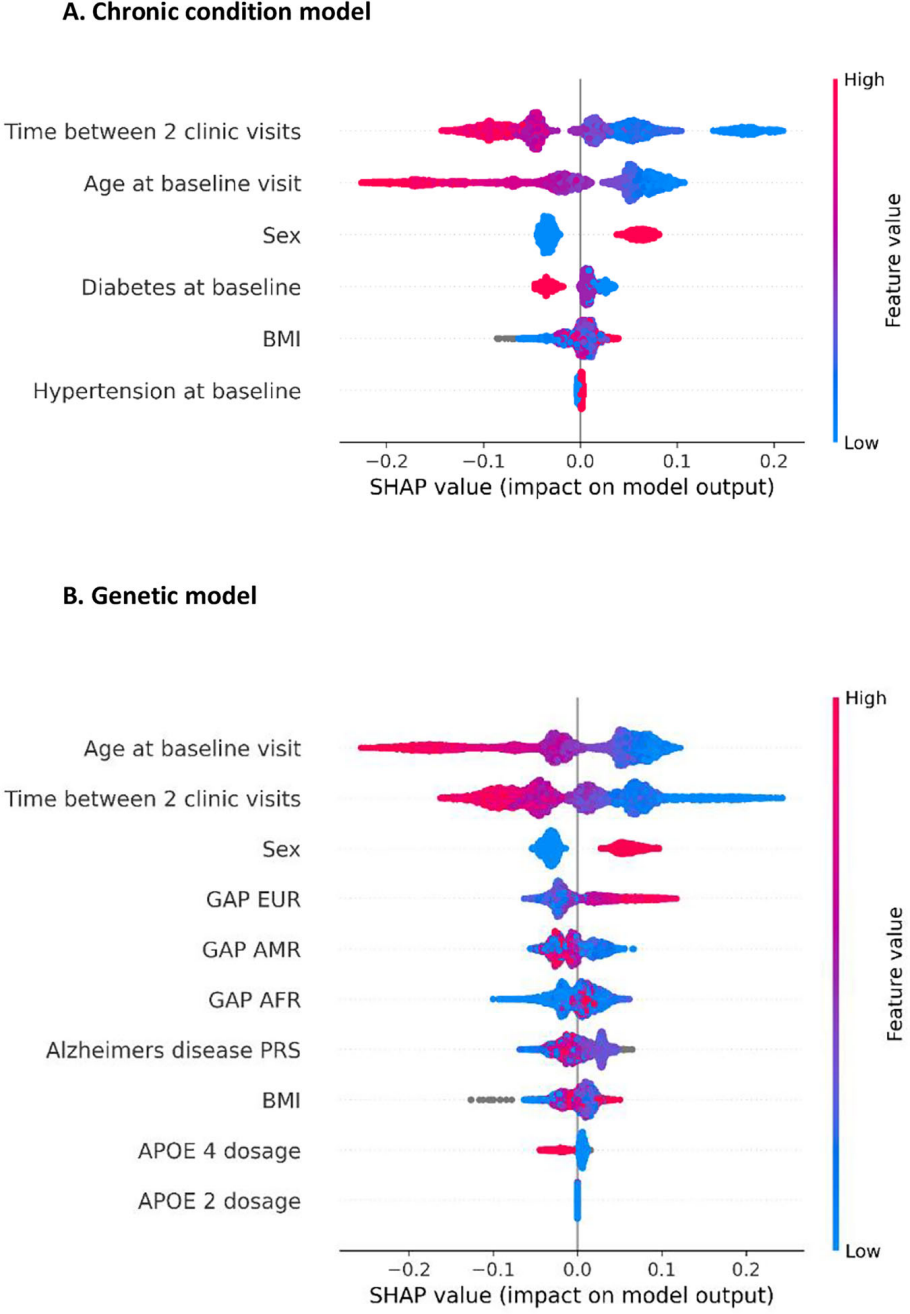
SHAP value plots for predicting GCSC based on selected models. Selected models included the chronic condition model (base + diabetes, hypertension; Panel **A**) and genetic model (Base + AD PRS, APOE alleles, GAPs; Panel **B**) using gradient-boosted trees. Features are listed in order of importance, from highest at the top of the plot to lowest at the bottom. Pink indicates high feature values and blue low feature values. SHAP values on the left indicate that the relevant feature contributed to a declining predicted GCSC and on the right contributed to improving the predicted GCSC. Sex male indicated by pink and sex female by blue. Having diabetes indicated by pink, pre-diabetes by purple, and not having diabetes by blue. Having hypertension indicated by pink and not having hypertension by blue.

**Fig. 5:**
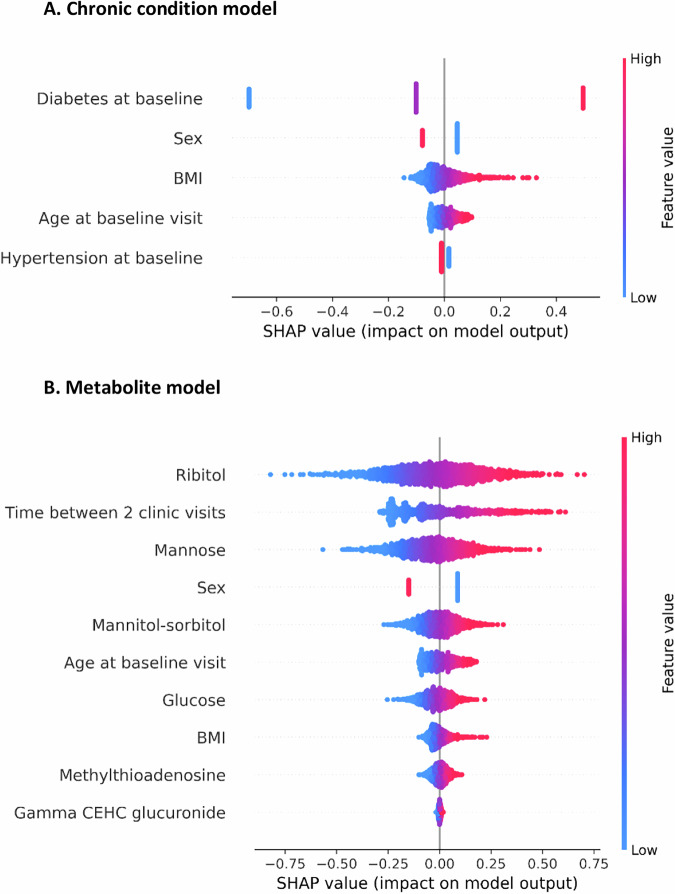
SHAP value plots for predicting MCI based on selected models. Selected models included the chronic condition model (base + diabetes, hypertension; Panel **A**) and metabolite model (Base + gamma-CEHC glucuronide, 5’-Methylthioadenosine, glucose, mannose, ribitol, and mannitol/sorbitol; Panel **B**) using logistic regression. Features are listed in order of importance, from highest at the top of the plot to lowest at the bottom. Pink indicates high feature values and blue low feature values. SHAP values on the left indicate that the relevant feature contributed to lowering prediction of MCI and on the right contributed to elevating prediction of MCI. Sex male indicated by pink and sex female by blue. Having diabetes indicated by pink, pre-diabetes by purple, and not having diabetes by blue. Having hypertension indicated by pink and not having hypertension by blue.

Based on our primary evaluation of model performance of the various combinations of predictor sets and methods, the chronic condition model, followed closely by the genetic model, using gradient-boosted trees appeared to predict GCSC slightly better than other models based on lowest mean MSE. For MCI, the chronic condition model, followed by the metabolite model, using logistic regression appeared to predict MCI slightly better based on highest mean AUC. Therefore, these models were selected to be fit in the overall dataset to evaluate feature impact using the Shapley Additive Explanations (SHAP) method. For gradient-boosted tree models of GCSC, the most important predictors in both the chronic condition and genetic predictor sets were time between visits, age at baseline, and sex, followed by diabetes in the chronic condition model and GAPs in the genetic model (Fig. 4). For logistic regression models of MCI, diabetes was the most important predictor in the chronic condition predictor set and ribitol in the metabolites predictor set (Fig. 5). In a secondary analysis combining diabetes and metabolites in a single predictor set, diabetes was the most important predictor followed by the metabolite ribitol (Supplementary Fig. 1).

For comparison with the SHAP based approach to ranking features, we also conducted secondary analyses to assess variable importance based on coefficient estimates and *p*-values from linear regression models based on the full model in the overall dataset. Reassuringly, these secondary analyses highlighted the same top features/variables as SHAP. Based on the linear regression model of GCSC in the overall dataset, significant variables included: time between visits (β = −0.92, SE = 0.015, *p* = 7.11 × 10^−10^); age at baseline (β = −0.011, SE = 0.002, *p* = 1.14 × 10^−6^); sex male (β = 0.123, SE = 0.033, *p* = 1.93 × 10^−4^); and GAP EUR (e.g., β = 0.286, SE = 0.077, *p* = 1.99 × 10^−4^) (Supplementary Table 7). Based on logistic regression model of MCI in the overall dataset, significant variables with *p* < 0.05 included: diabetes (β = 0.875, SE = 0.369, *p* = 0.02) and ribitol (β = 0.244, SE = 0.120, *p* = 0.04) (Supplementary Table 8).

## Discussion

In this paper we evaluated several prediction models of cognitive aging and impairment outcomes in Hispanics/Latinos using data from a large prospective cohort study in the U.S. We considered six predictor sets and two methods for GCSC and MCI. We evaluated six sets of predictors to assess the usefulness of administrating questionnaires or collecting data of a specific type (e.g., lifestyle vs genetic) in prediction of cognitive aging outcomes. We utilized two methods to assess whether non-linear machine learning methods improved performance compared to conventional linear methods. For non-linear models, we further incorporated cross validation and re-fitting of models into our study design to optimize tuning parameters necessary for gradient-boosted trees. Although predictive performance across models was not especially high and did not vary greatly by predictor sets and methods, we did glean helpful information to support and refine predictive models for GCSC and MCI, especially in Hispanic/Latino populations.

For GCSC, the mean MSE (95% CI) was similar across all combinations of methods and predictors sets, ranging from 0.2464 (0.2248–0.2684) to 0.2605 (0.2109–0.3055). Non-linear gradient-boosted trees appeared to slightly outperform traditional linear regression, with the chronic conditions model having slightly better performance, followed closely by the genetic model, than other predictor sets. Based on SHAP values, the most important factors in both models were time between visits, age at baseline, and sex. Interestingly, the base model which included these three factors plus BMI had slightly worse, though similar, performance (i.e. the mean MSE for the base model was higher than the mean MSE for the chronic condition and genetic models, but the confidence intervals for the models overlapped). Following time between visits, age at baseline and sex, the next most important factor(s) identified in the chronic condition model was diabetes and in the genetic model were GAPs. In particular, diabetes ranked higher than BMI, and GAPs ranked higher than genetic factors specific to cognition such as the AD PRS and *APOE*-$\epsilon$4 or *APOE*-$\epsilon$2 dosage. The lower ranking of *APOE*-$\epsilon$4 or *APOE*-$\epsilon$2 dosage is in line with previous research demonstrating a weaker association between the *APOE*-$\epsilon$4 gene allele and cognitive measures in Hispanics/Latinos compared to non-Hispanic Whites^10–13^, though further studies are needed to further characterize the contribution of *APOE*-$\epsilon$ variants to prediction models in diverse populations. Previous prediction models of cognitive impairment, as summarized in a review paper, often include age, sex, BMI and *APOE*-$\epsilon$4 carrier status^3^. Our results suggest that prediction models of GCSC in Hispanics/Latinos may benefit from incorporating information on diabetes and including ancestry in addition to *APOE*-$\epsilon$4 count or carrier status.

For MCI, the mean AUC (95% CI) was also similar across all combinations of methods and predictors sets, ranging from 54.33 (49.12–60.00) to 61.94 (51.21–70.24). Similar to GCSC, the chronic condition model again had slightly better performance for predicting MCI than other predictors, though logistic regression slightly outperformed gradient-boosted trees. Following the chronic condition model, the metabolite model had the next best performance in predicting MCI. Based on SHAP values, diabetes was the most important predictor in the chronic condition model and ribitol was most important in the metabolite model. In secondary analyses exploring models with both diabetes and metabolites, diabetes and ribitol continued to the most important predictors. Interestingly, both diabetes and ribitol appeared to be more important predictors of MCI than time between visits, age at baseline and sex. Indeed, the base model had slightly lower mean AUC than the chronic condition and metabolite models, though confidence intervals did overlap. Unlike GCSC, the genetic model was the worst performing model in predicting MCI. Surprisingly, the genetic model performed worse than even the base model; however, it is again important to note that the confidence intervals overlapped indicating that its performance may not be statistically different than the base model. Moreover, since logistic regression does not accommodate missing data, the poorer performance of the genetic model could be due to exclusion of those missing genetic data. The MCI results again support the utility of incorporating diabetes status into prediction modeling and further suggest that information on metabolites, particularly ribitol, could further enhance prediction of MCI in Hispanic/Latino populations.

It is not surprising that diabetes was an important predictor for both GCSC and MCI. Several previous studies on cognitive impairment have considered the role of diabetes^43,44^. In a large meta-analysis of 14 studies, covering 2,310,330 individuals, diabetes was associated with a 60% increased risk of dementia^45^. In another meta-analysis of 14 studies, covering 26,137 individuals, diabetes was associated with cognitive impairment in several domains, including motor function, executive functions, processing speed, verbal memory and visual memory^46^. Additionally, studies have also evaluated the severity of diabetes on cognitive impairment, suggesting that individuals with insulin resistance or elevated glucose levels not meeting the definition of diabetes may also be at increased risk for MCI or AD^47^. Among studies specific to Hispanic/Latino populations, who typically have higher prevalence of diabetes and ADRD than non-Hispanic White populations^1,48^, diabetes has been consistently associated with cognitive function in several studies^31–35^.

While most prior studies evaluating prediction models for cognitive measures have not incorporated information on diabetes, two studies did include diabetes in their prediction sets^3^. One study evaluated the predictive ability of the Australian National University AD Risk Index which includes age, sex, education level, diabetes, smoking, alcohol, and other health and lifestyle factors in three cohorts (Rush Memory and Aging Study, the Kungsholmen Project, and the Cardiovascular Health Cognition Study), finding AUCs ranging from 0.653 to 0.728 for any dementia and from 0.637 to 0.740 for AD^49^. A second study utilizing data from four large U.S. cohort studies (the Cardiovascular Health Study, the Framingham Heart Study, the Health and Retirement Study, and the Sacramento Area Latino Study on Aging) evaluated a prediction model including age, education level, stroke, diabetes, BMI, depressive symptoms, and assistance with money or medications, reporting accuracy ranging from 0.68 to 0.78, with the highest accuracy in SALSA a cohort of Hispanics/Latinos^50^. Although the chronic condition model in our study differed slightly from these previous models, our results and previous studies’ results support the utility of including diabetes status in prediction models of cognitive aging measures, particularly in Hispanic/Latino populations and especially since diabetes measures can easily be ascertained from questionnaires and/or clinical testing.

With respect to metabolites, the BPRHS (*N* = 736), noted 13 metabolites associated with a global cognitive score^37^. In a follow-up paper utilizing independent data from HCHS/SOL^36^, six of those metabolites replicated: gamma-CEHC glucuronide, 5’-Methylthioadenosine, glucose, mannose, ribitol, mannitol/sorbitol. Notably, these metabolites are predominantly carbohydrates and may therefore represent markers of diabetes or be casually involved in pathways linking diabetes and cognitive impairment^37^. Interestingly, ribitol appeared to be a more important predictor for MCI than glucose in the present study. Ribitol is a sugar alcohol formed by the reduction of ribose and high levels of ribitol have been observed in individuals with deficiency in enzymes in the pentose phosphate pathway (PPP)^51,52^. Two small previous studies comparing metabolomic profiles between cases of AD or MCI (*N* < 32) and healthy controls (*N* < 40) showed mixed evidence for an association between ribitol and cognitive outcomes^53,54^. Previously we noted some evidence of a potential causal relationship between ribitol and cognitive function based on Mendelian Randomization^36^. Three previous studies evaluated prediction models of cognitive aging specifically among individuals with diabetes, with some considering measures of glucose, but no studies considered metabolites such as ribitol^3,55–57^. While including metabolite measures, such as ribitol, may help improve prediction of some cognitive measures, such as MCI, it is important to consider that the slight improvement in performance may not justify the additional cost and time to collect metabolite data.

Beyond the predictor set, we noted some evidence that non-linear gradient-boosted trees may outperform conventional linear regression for GCSC prediction which may reflect the utility of gradient-boosted trees to incorporate multiple types of data, particularly genetic and environmental factors, into prediction models among diverse populations^39^. In particular, gradient-boosted trees have the potential to improve outcome prediction when relationships between predictors and outcomes are complex and may differ by subgroups of populations, which is possible when studying numerous types of predictors and GCSC among Hispanics/Latinos. Another advantage of gradient-boosted trees is that it accommodates missing values thereby increasing sample size, while linear regression requires complete-case analysis. When we restricted gradient-boosted trees models to individuals with complete data in secondary analyses, the models no longer outperformed linear regression models suggesting that the increased sample size resulting from gradient-boosted trees ability to incorporate missing data was important to its improved performance. Furthermore, when we stratified by age, linear regression performed better than gradient-boosted trees for predicting GCSC. Therefore, it is possible that the gradient-boosted trees model captured some non-linearity that is present when the age groups are aggregated. Finally, we found that logistic regression performed better for MCI. This could reflect less variation in a binary compared to continuous outcome.

Since previous ADRD prediction studies were largely conducted in populations of European ancestry, our study extends efforts to predict ADRD-precursor conditions by assessing GCSC and MCI in a large population of Hispanics/Latinos. Our comprehensive approach which included consideration of multiple models incorporating lifestyle, chronic conditions, genetics and metabolomic factors and both linear and non-linear methods was also a strength. Rather than using stepwise regression which does not account for innate grouping of variables and is sensitive to the order in which variables are added or removed from the model, we constructed and compared models based on natural groupings of risk factors previously associated with cognitive aging measures in order to determine which types of data (e.g. questionnaire data on lifestyle variables or biologic data on genetics or metabolomics) are most important for prediction modeling. Additionally, splitting our data into training and testing sets allowed us to carefully assess models defined by the combinations of predictors and methods, and performing five-fold cross validation allowed us to identify optimal values for tuning parameters.

Our study also has a few weaknesses. Despite our large sample size, there was a limited number of individuals with MCI, especially after excluding individuals without genetic or metabolomic data. The average age of our population at baseline (55 years) and average follow-up time (7 years) may have also contributed to the limited number of individuals with MCI. Further, participation and reporting accuracy can depend on cognitive ability. Although we are not able to assess whether cognitive decline prior to baseline impacted enrollment nor reporting accuracy, we were able to explore whether cognitive ability at baseline impacted participation at follow-up. The slightly higher missing rate among those with “poor” baseline GCS may have attenuated GCSC results and also contributed to the limited number of MCI cases. Although model performance varied slightly by age, it is important to note that the same predictor sets identified as having slightly better performance in the primary analyses were also highlighted in the age-stratified analyses.

Other limitations include that global cognitive score change was based on just two visits which may be noisier than if measures were based on several visits. The six metabolites considered in our prediction models were chosen based on previous work that included HCHS/SOL, though the metabolites were originally identified in an external study (BPRHS) for a different cognitive measure (global cognitive function rather than MCI)^37^. While we were able to examine well-known APOE variants (i.e. APOE-ϵ4 or APOE-ϵ2) in our prediction models, we were not able to consider rare protective APOE variants, in particular APOE-Christchurch which is nearly monomorphic in Admixed Americans (frequency based on gnomAD/Ensembl databases is only 0.0001)^58^. Finally, although the AD PRS was based on multiethnic GWAS results, the majority of individuals in GWAS were of European ancestry which may limit prediction utility in populations of Hispanic/Latinos. Few Hispanic/Latino individuals have been included in previous AD GWAS which inhibits our ability to consider a Hispanic/Latino specific PRS. As GWAS start to include more Hispanic/Latino individuals and methods for constructing multiethnic PRS (e.g., PRS-CSx) improve, constructing AD PRS more tailored to Hispanic/Latino individuals could improve prediction utility.

In summary, we assessed several potential prediction models for cognitive aging outcomes among a Hispanic/Latino population. Since GCSC and MCI can be harbingers of dementia, accurately predicting such precursor conditions is important for early intervention. For both GCSC and MCI, performance was similar across all predictors sets, though the chronic condition model seemed to perform slightly better. In particular, diabetes appeared to be an important predictor for both outcomes. In addition to demographics (age and sex) and diabetes, genetic ancestry appeared to be important for GCSC and metabolites, namely ribitol, appeared to be important for MCI. Diabetes status is easily obtained through questionnaire or simple clinical testing, and therefore represents an important and implementable addition to prediction modeling of cognitive aging measures, especially in Hispanic/Latino populations. In contrast, genetic and metabolite measures require more resources which can limit their utility in prediction modeling. While logistic regression performed slightly better for MCI, gradient-boosted trees slightly outperformed linear regression for GCSC. This may reflect that traditional modeling approaches may be sufficient for binary outcomes such as MCI and its associated predictor sets, while gradient-boosted trees which accommodate complex data structures and allow for missing data may be warranted for continuous outcomes such as GCSC and its associated predictor sets. Although prediction performance was not especially high and did not vary greatly across models, incorporating information on diabetes, ancestry and metabolites may help improve prediction of GCSC and MCI in Hispanic/Latino populations. As longitudinal data from the SOL-INCA aging population is added, and genomics measures that are stronger predictors of ADRD and of cognitive decline become available, future work should continue to evaluate the potential for early prediction of cognitive aging.

## Methods

### Study population

HCHS/SOL is a longitudinal study of 16,415 individuals of self-reported Hispanic/Latino origin (Cuban, Puerto Rican, Dominican, Mexican, and Central/South American)^59,60^. Participants were aged 18–74 years at baseline, with oversampling for individuals aged 45–74 years. A two-stage probability sampling design based on census-blocks groups and household addresses was used to enroll individuals from four geographic sites in the U.S. (Miami, San Diego, Chicago, and the Bronx area of New York). Participants completed comprehensive clinical examinations and health questionnaires at baseline (2008–2011) and during a follow-up visit (2014–2017). In conjunction with the HCHS/SOL visit 2, an ancillary study, SOL-INCA, occurred to repeat and expand the baseline battery of cognitive measures^61^.

The HCHS/SOL was approved by the institutional review boards (IRBs) at each field center, where all participants gave written informed consent, and by the Non-Biomedical IRB at the University of North Carolina at Chapel Hill, to the HCHS/SOL Data Coordinating Center. All IRBs approving the HCHS/SOL study are: Non-Biomedical IRB at the University of North Carolina at Chapel Hill. Chapel Hill, NC; Einstein IRB at the Albert Einstein College of Medicine. Bronx, NY; IRB at Office for the Protection of Research Subjects (OPRS), University of Illinois at Chicago. Chicago, IL; Human Subject Research Office, University of Miami. Miami, FL; Institutional Review Board of San Diego State University, San Diego, CA. All methods and analyses of HCHS/SOL participants’ materials and data were carried out in accordance with human subject research guidelines and regulations. This work was approved by the Beth Israel Deaconess Medical Center Committee on Clinical Investigations (2023P000346). Clinical trial number: not applicable.

### Lifestyle data

Information on sleep duration, diet, and exercise were obtained via questionnaires administered at the baseline visit. Sleep duration questions included: “What time do you usually go to bed (on weekdays, on weekends)?” and “What time do you usually wake up (on weekdays, on weekends)?”^18^. An overall measure of sleep duration was calculated as a weighted average of sleep duration on weekdays and weekends. Diet was ascertained through a 24-h dietary recall and used to calculate a Mediterranean diet score (MDS) which ranges from 0 to 9 based on consumption of fruits, vegetables, legumes, cereals, fish, meat, dairy products, alcohol, and the monounsaturated to saturated fat ratio^14^. Information on exercise was collected using the Global Physical Activity Questionnaire (GPAQ) which queried how many days and minutes each day respondents spent doing moderate or vigorous activities at work or for recreation (such as sports or fitness) as well as for transport (such as walking and cycling)^62,63^. An overall measure of exercise was then calculated by summing work-related physical activity (min/day), recreational physical activity (min/day), and transportation-related physical activity (min/day).

### Chronic conditions data

Clinical examinations, including fasting blood draw and blood pressure measurement, was completed at baseline. Diabetes at baseline was defined based on the American Diabetes Associations guidelines using information on fasting glucose (mg/dL), post OGGT glucose (mg/dL), % glycosylated hemoglobin (A1C), elapsed time (hours) between the time the participant last consumed anything and the blood draw, and anti-diabetes medication use^64^. Individuals were classified as having diabetes if fasting glucose was ≥126 mg/dL and fasting time was >8 h, fasting glucose was ≥200 mg/dL and fasting time was ≤8 h, post-OGTT glucose was ≥200 mg/dL, A1C was ≥6.5%, or use of anti-diabetes medication. Individuals were classified as having pre-diabetes if fasting glucose ranged from 100 to 125 mg/dL and fasting time was >8 h, post-OGTT glucose ranged from 140 to 199 mg/dL, or AC1 ranged from 5.7 to 6.5%. Individuals not meeting the criteria for diabetes or pre-diabetes were classified as non-diabetes. Hypertension at baseline was defined based on the American Heart Association guidelines as systolic blood pressure (SBP) ≥ 130, diastolic blood pressure (DBP) ≥ 80 or use of hypertension medication^65^.

### Metabolomic data

As previously described^66^, metabolites were quantified in fasting serum using the discovery HD4 platform (Metabolon Inc Durham NC) in two “batches”. In the first batch, which was profiled in 2017, serum samples from 4002 individuals were assayed, with individuals sampled at random from HCHS/SOL individuals who had whole-genome sequencing data at the time. The second batch, which was profiled in 2021, had samples from 2306 participants, including individuals overlapping with batch 1 to facilitate harmonization of the data, and additional individuals selected based on criteria of particular HCHS/SOL sub-studies, which overrepresented older individuals and persons with reduced estimated glomerular filtration rate. Consequently, batch 2 participants tend to be a bit older, on average, than batch 1 participants. The data were corrected for technical effects by Metabolon Inc. Principal components analysis of metabolites by batch revealed no batch effects (Supplementary Fig. 2). Therefore, additional batch correction methods such as ComBat were not employed. Instead, the two batches were harmonized as follows. First, we required that a metabolite have no more than 20% missing values in a given batch. For included metabolites, we set missing values to half the minimum value observed for that metabolite in the batch, assuming that missingness is due to the value being below the limit of detection. Next, the values of each metabolite were rank-normalized within each batch. Individuals from batch 2 who overlapped with batch 1 were removed, and repeated samples within each batch were selected at random to keep one sample per individuals. Overall, there were 6180 individuals in the batch-combined dataset. In previous cross-sectional analyses of HCHS/SOL, ARIC and the BPRHS, six metabolites were found to be consistently associated with lower cognitive function^36,37^: gamma-CEHC glucuronide, 5’-Methylthioadenosine, glucose, mannose, ribitol, and mannitol/sorbitol. Therefore, the present analysis evaluated inclusion of these six metabolites in prediction models.

### Genetic data

Individuals consenting to genetic studies had blood drawn at baseline^5,67^. Genotyping was performed using the Illumina HumanOmni2.5 BeadChip array. Variants with >5% missingness or MAF < 0.005 and individuals with >5% missingness across all SNPs were removed from analysis. Genome-wide imputation was then completed using the University of Michigan imputation server based on the Trans-Omics in Precision Medicine (TOPMed) 2.0 reference panel. Variants with R^2^ ≤ 0.4 or MAF < 0.01 after imputation were discarded. Global ancestry proportions for the HCHS/SOL ancestral populations, namely European (EUR), African (AFR), and Indigenous American (AMR), were estimated based on genotyping using the ADMIXTURE software as previously described^5^. Briefly, GAPs were calculated in two stages. First, an unsupervised ADMIXTURE analysis was performed using reference-population samples from the Human Genome Diversity Project (HGDP) and identified 101 African, 49 Amerindian, and 176 European individuals with estimated ancestry >90% from the respective group (initially, East Asian reference individuals were also included but they were later excluded in re-analysis using three reference populations, rather than four). Next, these selected HGDP individuals were used as a reference sample in an application of a supervised ADMIXTURE analysis applied over unrelated HCHS/SOL individuals (kinship coefficient <0.044 for all pairs, identified using the PC-AiR algorithm. These analyses were performed using 92,992 autosomal single nucleotide polymorphisms (SNPs) in common to the HGDP reference individuals and to HCHS/SOL, and that were selected by linkage disequilibrium (LD) pruning. As documented in previous publications^5,6,68–70^, it is anticipated that individuals who self-identify as Hispanic/Latino will have varying levels of African, European and Indigenous American ancestry. Therefore, we did not exclude individuals based on any GAPs thresholds. *APOE* genotyping was performed separately using a TaqMan assay^12,71^. A polygenic risk score (PRS) for Alzheimer’s disease (AD) was constructed based on the multi-ethnic AD PRS developed in Sofer et al.^13^. Briefly, the multi-ethnic AD PRS was constructed by summing study-specific PRS developed from each of the following GWAS: Kunkle et al. 2019 (63,926 individuals of European ancestry)^72^, Kunkle et al. 2021 (8006 individuals of African ancestry)^73^, Bellenguez et al. (487,511 individuals of European ancestry)^74^, FINNGEN (342,499 Finnish individuals)^75^, Jun et al. (33,269 individuals of European, African, Japanese, and Israeli ancestry)^76^, and Lake et al. (644,188 individuals of European, African, East Asian, and Caribbean Hispanic ancestry)^77^. PRS weights were available from the PGS catalog, PGS ID PGS003958.

### Cognitive data

Participants 45 years of age or older who consented were administered cognitive tests at baseline in HCHS/SOL (*N* = 9652) and at follow-up in SOL-INCA, an ancillary study occurring an average of 7 years after baseline (*N* = 6377)^61,78^. In this analysis, two cognitive aging outcomes were considered: global cognitive score change (GCSC) and MCI. Cognitive tests at baseline and follow-up included a Spanish English Verbal Learning Test, a Spanish English verbal episodic learning and memory test, a word frequency test, and a digit symbol substitution test. Additionally, trail making tests were administered at follow-up^61^. From these tests we computed composite global cognitive scores (GCS) at baseline and follow-up. Specifically, we estimated sample means and standard deviations (SDs) for each test at the corresponding visit using the survey package to account for the target population. We then standardized each individual’s test-specific scores at baseline and follow-up by subtracting the test- and visit-specific mean and dividing by the corresponding SD to obtain z-scores. Test-specific z-scores at each visit were then averaged to obtain a composite score for each participant at baseline and follow-up. Finally, GCSC was calculated as the difference in GCS at follow-up and GCS at baseline, with negative values indicating a decline in cognitive health, positive values indicating learning effects or improvements in cognition and larger magnitude values implying greater change. MCI at follow-up (SOL-INCA) was defined as a binary outcome (yes vs no) based on meeting all three of the following National Institute on Aging-Alzheimer’s Association criteria: 1) a cognitive test score below –1 SD based on robust internal norms, (2) significant cognitive decline exceeding –0.055 standard deviation (SD) yearly based on a latent factor model, (3) significant self-reported subjective cognitive decline based on the E-Cog12 and no or minimal limitations in activities of daily living^12,61,79^.

### Development, evaluation and explanation of prediction models for cognitive aging outcomes

The framework for developing the prediction models consisted of several phases including: 1) Evaluation phase, where we combined different sets of potential predictors and methods to evaluate model performance for each combination; 2) Selection phase, where we selected a model (i.e. combination of predictor set and prediction method) for each of the two outcomes, GCSC and MCI, based on average model performance metrics; and 3) Explanation phase, where we estimated each feature’s contribution to the prediction of the selected model. A detailed visual of this framework is presented in Fig. 1.

In the Evaluation phase (Fig. 1B) six predictor sets of varying complexity were considered: 1) Base (age, sex, BMI, time from baseline to follow-up), 2) Genetic (base + *APOE*-$\epsilon$2 and *APOE*-$\epsilon$4 variant dosage, AD PRS, GAPs for AFR, EUR and AMR), 3) Lifestyle (base + sleep duration, Mediterranean diet score, exercise score), 4) Metabolites (base + gamma-CEHC glucuronide, 5’-Methylthioadenosine, glucose, mannose, ribitol, mannitol/sorbitol), 5) Chronic Condition (base + diabetes, hypertension) and 6) Full (base + genetic + lifestyle + metabolites + chronic conditions). Supplementary Table 9 summarizes the rationale for evaluating each predictor set. Linear regression (LR) was used to model GCSC continuously and logistic regression was used to model MCI (yes vs no). We also modeled outcomes using gradient-boosted trees implemented with the LightGBM package in Python to evaluate whether non-linear machine learning methods performed better for predicting GCSC and MCI.

To robustly measure model performance, the data were split into a testing set (~20% of participants) and training set using random seeds over 100 repetitions (Fig. 1B). Due to strong imbalance in the dataset (~90% of participants were not classified with MCI), we split the dataset based on the MCI variable to ensure an approximately equal proportion of MCI cases in the train and test sets. For models using gradient-boosted trees, we tuned hyperparameters using the Python Optuna library (version 3.0.6)^80^. Specifically, we randomly divided the training dataset into five independent subsets and applied a 5-fold cross-validation process to identify the optimal values for the relevant hyperparameters which were then integrated into each model during each of the 100 repetitions (Fig. 1C).

For GCSC prediction, model performance was evaluated using MSE which measures the difference between the predicted value and actual value and therefore lower values indicate better performance. MSE was calculated using the Python *sklearn.metrics* library. For MCI prediction, model performance was evaluated using AUC which measures the likelihood that the model will assign a higher probability to MCI classification than to non-MCI classification, with higher values indicating better performance. Mean MSE or AUC were calculated across the 100 test sets, with 95% confidence intervals defined as the empirical 2.5 and 97.5 percentile interval. Differences in MSE and AUC between select models and the base model were also calculated and averaged across the 100 test sets. Additional model performance metrics such as accuracy and the F1 score, a measure of precision and recall, were also noted.

During the Selection phase, the model (defined by the combination of predictor set and modeling method) with the best average performance metric across the 100 iterations was then selected and fit on the full dataset (Fig. 1D). Finally in the Explanation phase, we computed Shapley additive explanation (SHAP) values using the Python *shap* library to identify the predictors with the highest contribution to the model’s prediction (Fig. 1D)^81^.

We also performed the following sensitivity analyses. To explore whether cognitive ability at baseline impacted participation at follow-up, we classified age-adjusted baseline GCS as “poor” if greater than 1 SD below the median and then compared the frequency of individuals missing follow-up GCS among those with and without “poor “baseline GCS. Additionally, to assess whether prediction model performance varied by age, we evaluated predictions models within strata of individuals aged ≤55 and >55 years old. While linear regression models require datasets without missing values, gradient-boosted trees have the advantage of being able to make predictions even with missing data. Therefore, to achieve the same sample size across methods and facilitate more direct comparisons of performance, we additionally ran gradient-boosted trees excluding missing data. Finally, because several of the metabolites associated with cognitive function in previous papers and considered in this paper were carbohydrates, we also fit the metabolite model including diabetes as a predictor.

All data preprocessing analyses were conducted in R and model prediction analyses were done using Python.

## Supplementary information

Supplemental Table

## Data Availability

The HCHS/SOL fully supports data sharing for HCHS/SOL–approved manuscript proposals with outside investigators. All data sharing is conducted in accordance with HCHS/SOL study and NIH policies and governed by a Data and Materials Distribution Agreement (DMDA) between UNC and the external institution, ensuring the confidentiality and privacy of HCHS/SOL participants and their families. Alternatively, de-identified HCHS/SOL data are publicly available at BioLINCC and dbGaP for the subset of the study cohort who authorized general use of their data at the time of informed consent. HCHS/SOL genotype and phenotype data are also available from dbGaP according to the study-specific accession “phs000810”.

## Code availability

Example analysis code available at: https://github.com/tamartsi/Cognitive_aging_prediction_SOL-INCA.
