# Supplementary material for: An integrated lifestyle-, genetic- and metabolomics-based prediction model for mild cognitive aging in the HCHS/SOL
Source: NPJ Dement. 2026 Jul 8;2(1):58. doi: 10.1038/s44400-026-00120-9 (PMC13345926; doi:10.1038/s44400-026-00120-9)
Supplement: Supplementary file 1 — Supplemental Table [file 44400_2026_120_MOESM1_ESM.pdf]

Supplemental Table 1. Model Prediction Performance for GCSC, Average MSE Across 100 Test Splits

	MSE (95% CI)					
**Models***	**Linear Regression**		**Gradient-Boosted Trees**		**Gradient-Boosted Trees, No Missing Data**	
**Base**	0.2595	0.2099, 0.3062	0.2471	0.2263, 0.2702	0.2628	0.2135, 0.3096
**Genetic**	0.2605	0.2109, 0.3055	0.2464	0.2244, 0.2708	0.2633	0.2132, 0.3103
**Lifestyle**	0.2601	0.2110, 0.3058	0.2474	0.2274, 0.2697	0.2639	0.2201, 0.3141
**Metabolites**	0.2595	0.2109, 0.3046	0.2474	0.2252, 0.2697	0.2656	0.2203, 0.3206
**Chronic Conditions**	0.2586	0.2110, 0.3054	0.2464	0.2248, 0.2684	0.2615	0.2102 0.3125
**Full**	0.2591	0.2106, 0.3061	0.2475	0.2255, 0.2847	0.2638	0.2207, 0.3193

*The six models included: 1) Base (age, sex, BMI, time between exams), 2) Genetic (Base + APOE alleles, AD PRS), 3) Lifestyle (Base + sleep duration, Mediterranean diet score, exercise), 4) Metabolites (Base + gamma-CEHC glucuronide, 5'-Methylthioadenosine, glucose, mannose, ribitol, and mannitol/sorbitol), and 5) Chronic Conditions (Base + diabetes, hypertension), 6) Full (base + genetic + lifestyle + metabolites+ chronic conditions)

Supplemental Table 2. Difference in Selected Model Prediction Performance for GCSC, Average Difference in MSE Across 100 Test Splits

		GCSC Gradient Boosted Trees MSE			Difference in MSE	
**Analysis**	**Mean (95% CI)**	**Base**	**Chronic Condition**	**Genetic**	**Chronic Condition - Base**	**Genetic - Base**
**Primary Age Combined**	**Mean**	0.2471	0.2464	0.2464	-6.94E-04	-6.71E-04
	**95% CI Lower**	0.2263	0.2248	0.2244	-9.12E-03	-8.54E-03
	**95% CI upper**	0.2702	0.2684	0.2708	5.72E-03	7.84E-03
		**GCSC Linear Regression MSE**				
**Age ≤ 55 years**	**Mean**	0.2061	0.2067	0.2088	0.0007	0.0027
	**95% CI Lower**	0.1443	0.1447	0.1472	-0.0015	-0.0025
	**95% CI upper**	0.2736	0.2728	0.2747	0.0042	0.0111
**Age > 55 years**	**Mean**	0.2578	0.2576	0.2532	-0.0002	-0.0045
	**95% CI Lower**	0.1916	0.1932	0.1935	-0.0068	-0.0179
	**95% CI upper**	0.3260	0.3288	0.3316	0.0097	0.0140

*Selected models included: 1) Base (age, sex, BMI, time between exams), 2) Genetic (Base + APOE alleles, AD PRS), and 5) Chronic Conditions (Base + diabetes, hypertension)

Supplemental Table 3. Model Prediction Performance for GCSC Among Individuals ≤55 or >55 Years Old, Average MSE Across 100 Test-Train Split

Models*	MSE (95% CI)							
	Linear Regression				Gradient-Boosted Trees			
	Age ≤ 55		Age > 55		Age ≤ 55		Age > 55	
**Base**	0.2061	0.1443, 0.2736	0.2578	0.1916, 0.3260	0.2328	0.2101, 0.2623	0.2622	0.2313, 0.2936
**Genetic**	0.2088	0.1472, 0.2747	0.2532	0.1935, 0.3316	0.2322	0.2100, 0.2637	0.2619	0.2307, 0.2928
**Lifestyle**	0.2074	0.1465, 0.2747	0.2558	0.1933, 0.3266	0.2327	0.2089, 0.2619	0.2626	0.2320, 0.2948
**Metabolites**	0.2079	0.1477, 0.2743	0.2589	0.2007, 0.3329	0.2325	0.2088, 0.2622	0.2631	0.2329, 0.2934
**Chronic Conditions**	0.2067	0.1447, 0.2728	0.2576	0.1932, 0.3288	0.2328	0.2099, 0.2620	0.2619	0.2306, 0.2921
**Full**	0.2126	0.1493, 0.2794	0.2567	0.1954, 0.3348	0.2322	0.2112, 0.2619	0.2621	0.2305, 0.2935

*The six models included: 1) Base (age, sex, BMI, time between exams), 2) Genetic (Base + APOE alleles, AD PRS), 3) Lifestyle (Base + sleep duration, Mediterranean diet score, exercise), 4) Metabolites (Base + gamma-CEHC glucuronide, 5'-Methylthioadenosine, glucose, mannose, ribitol, and mannitol/sorbitol), and 5) Chronic Conditions (Base + diabetes, hypertension), 6) Full (base + genetic + lifestyle + metabolites+ chronic conditions)

Supplemental Table 4. Model Prediction Performance for MCI, Average AUC Across 100 Test-Train Splits

	Logistic Regression				Gradient-Boosted Trees				Gradient-Boosted Trees, No Missing Data			
**Models***	**AUC (95% CI)**		**Accuracy**	**F1**	**AUC (95% CI)**		**Accuracy**	**F1**	**AUC (95% CI)**		**Accuracy**	**F1**
**Base**	0.5760	0.4836, 0.6944	0.5819	0.1990	0.5504	0.5000, 0.6110	0.8968	0.1207	0.5011	0.4645, 0.5511	0.8948	0.1787
**Genetic**	0.5502	0.4591, 0.6606	0.5757	0.1865	0.5433	0.4912, 0.6000	0.8857	0.1133	0.4964	0.4110, 0.5662	0.8847	0.0905
**Lifestyle**	0.5698	0.4761, 0.6587	0.5821	0.2040	0.5550	0.4938, 0.6194	0.8870	0.1096	0.4986	0.4350, 0.5620	0.8898	0.1513
**Metabolites**	0.5980	0.4920, 0.7048	0.5977	0.2078	0.5613	0.5065, 0.6142	0.8903	0.1348	0.5707	0.4665, 0.6655	0.8746	0.1483
**Chronic Conditions**	0.6194	0.5121, 0.7024	0.6009	0.2170	0.5980	0.5366, 0.6442	0.8913	0.1250	0.5271	0.4809, 0.6518	0.8784	0.1895
**Full**	0.5946	0.4779, 0.6935	0.6069	0.2080	0.5764	0.5254, 0.6372	0.8751	0.1168	0.5839	0.4911, 0.7090	0.862	0.1400

*The six models included: 1) Base (age, sex, BMI, time between exams), 2) Genetic (Base + APOE alleles, AD PRS), 3) Lifestyle (Base + sleep duration, Mediterranean diet score, exercise), 4) Metabolites (Base + gamma-CEHC glucuronide, 5’-Methylthioadenosine, glucose, mannose, ribitol, and mannitol/sorbitol), 5) Chronic Conditions (Base + diabetes, hypertension), 6) Full (base + genetic + lifestyle + metabolites+ chronic conditions)

Supplemental Table 5. Difference in Selected Model Prediction Performance for GCSC, Average Difference in AUC Across 100 Test Splits

		MCI Logistic AUC			Difference in AUC	
**Analysis**	**Mean (95% CI)**	**Base**	**Chronic Condition**	**Metabolite**	**Chronic Condition - Base**	**Metabolite - Base**
**Primary Age Combined**	**Mean**	0.5760	0.6194	0.5980	0.0435	0.0220
	**95% CI Lower**	0.4836	0.5121	0.4920	-0.0485	-0.0867
	**95% CI upper**	0.6944	0.7024	0.7048	0.1324	0.1267
**Age ≤ 55 years**	**Mean**	0.5207	0.5688	0.5832	0.0481	0.0624
	**95% CI Lower**	0.457	0.5092	0.4374	-0.0288	-0.1153
	**95% CI upper**	0.5855	0.6428	0.7185	0.1177	0.2189
**Age > 55 years**	**Mean**	0.5855	0.6032	0.5927	0.0177	0.0072
	**95% CI Lower**	0.5075	0.5291	0.4501	-0.0143	-0.1482
	**95% CI upper**	0.6605	0.6694	0.7065	0.0423	0.1640

[*]Selected models included: 1) Base (age, sex, BMI, time between exams), 4) Metabolites (Base + gamma-CEHC glucuronide, 5’-Methylthioadenosine, glucose, mannose, ribitol, and mannitol/sorbitol), and 5) Chronic Conditions (Base + diabetes, hypertension)

Supplemental Table 6. Model Prediction Performance for GCSC Among Individuals ≤55 or >55 Years Old, Average AUC Across 100 Test-Train Split

Models*	AUC (95% CI)							
	Logistic Regression				Gradient-Boosted Trees			
	Age ≤ 55		Age > 55		Age ≤ 55		Age > 55	
**Base**	0.5207	0.4570, 0.5855	0.5855	0.5075, 0.6605	0.4948	0.4191, 0.5580	0.5587	0.4837, 0.6196
**Genetic**	0.4917	0.4055, 0.5733	0.5787	0.5099, 0.6524	0.4821	0.4170, 0.5575	0.5633	0.4937, 0.6288
**Lifestyle**	0.5195	0.4386, 0.5847	0.5777	0.5094, 0.6577	0.5354	0.4729, 0.6209	0.5565	0.4913, 0.6191
**Metabolites**	0.5832	0.4374, 0.7185	0.5927	0.4501, 0.7065	0.5085	0.4391, 0.5630	0.5678	0.4988, 0.6363
**Chronic Conditions**	0.5688	0.5092, 0.6428	0.6032	0.5291, 0.6694	0.5563	0.4783, 0.6456	0.5883	0.5313, 0.6496
**Full**	0.5510	0.4031, 0.6900	0.6074	0.4674, 0.7270	0.5682	0.5034, 0.6546	0.5813	0.5191, 0.6426

*The six models included: 1) Base (age, sex, BMI, time between exams), 2) Genetic (Base + APOE alleles, AD PRS), 3) Lifestyle (Base + sleep duration, Mediterranean diet score, exercise), 4) Metabolites (Base + gamma-CEHC glucuronide, 5'-Methylthioadenosine, glucose, mannose, ribitol, and mannitol/sorbitol), and 5) Chronic Conditions (Base + diabetes, hypertension), 6) Full (base + genetic + lifestyle + metabolites+ chronic conditions)

Supplemental Table 7. Coefficient Estimates and P-values from Linear Regression of GCSC based on Full Model in Combined (Train+Test) Dataset

Variable	Estimate	Std.Error	Z	Pvalue
Intercept	1.056	0.215	4.915	1.02E-06
Years Between Visits	-0.092	0.015	-6.216	7.11E-10
Age at Baseline	-0.011	0.002	-4.892	1.14E-06
Sex (male)	0.123	0.033	3.740	1.93E-04
GAP EUR	0.286	0.077	3.732	1.99E-04
Hypertension	0.053	0.034	1.579	0.11
Glucose	-0.029	0.025	-1.157	0.25
Sleep Duration	-0.011	0.011	-1.079	0.28
APOE4 Count	-0.036	0.034	-1.063	0.29
Diabetes	-0.050	0.052	-0.965	0.33
APOE2 Count	0.045	0.050	0.905	0.37
Mediterranean Diet Score	-0.007	0.010	-0.783	0.43
Mannitol/Sorbitol	-0.012	0.018	-0.677	0.50
Methylthioadenosine	-0.010	0.017	-0.585	0.56
Total Physical Activity	-9.53E-06	1.88E-05	-0.507	0.61
BMI	0.001	0.003	0.451	0.65
Pre-diabetes	0.016	0.038	0.418	0.68
Mannose	0.008	0.022	0.337	0.74
GAP AFR	0.021	0.104	0.201	0.84
AD PRS (scaled)	0.003	0.017	0.174	0.86
Gamma CEHC Glucuronide	0.001	0.016	0.080	0.94
Ribitol	-6.19E-04	0.017	-0.036	0.97

Supplemental Table 8. Coefficient Estimates and P-values from Logistic Regression of MCI based on Full Model in Combined (Train + Test) Dataset

Variable	Estimate	Std.Error	Z	Pvalue
Intercept	-4.417	1.434	-3.080	0.002
Diabetes	0.875	0.369	2.369	0.02
Ribitol	0.244	0.120	2.030	0.04
Years Between Visits	0.183	0.095	1.922	0.05
Mediterranean Diet Score	-0.081	0.065	-1.244	0.21
Pre-diabetes	0.360	0.311	1.158	0.25
Sleep Duration	0.068	0.070	0.968	0.33
GAP AFR	-0.602	0.721	-0.835	0.40
APOE4 Count	0.184	0.220	0.835	0.40
Mannose	0.129	0.157	0.818	0.41
GAP EUR	-0.410	0.508	-0.808	0.42
Mannitol/Sorbitol	0.100	0.124	0.807	0.42
Glucose	-0.131	0.173	-0.756	0.45
Age at Baseline	0.008	0.015	0.581	0.56
Total Physical Activity	0.000	0.000	-0.533	0.59
Sex (male)	-0.092	0.228	-0.403	0.69
APOE2 Count	0.105	0.346	0.302	0.76
BMI	0.003	0.018	0.171	0.86
Gamma CEHC Glucuronide	-0.012	0.106	-0.111	0.91
AD PRS (scaled)	0.010	0.117	0.085	0.93
Methylthioadenosine	0.003	0.117	0.023	0.98
Hypertension	1.98E-04	0.234	0.001	1.00

Supplemental Table 9. Summary of Predictor Sets and Rationale

Predictor Set	Variables	Rationale
Base	Age, Sex, BMI, Time from baseline to follow up (SOL-INCA)	Basic demographic variables standard in previous prediction models of cognitive measures[1]
Genetic Cognitive	Base + *APOE*-ϵ2 and *APOE*-ϵ4 variant dosage, AD PRS, global ancestry proportions for AFR, EUR and AMR	*APOE* variants are a well-known risk factor cognitive measures and often included in previous prediction models; however, this association may vary in Hispanics/Latinos[2,3]. Previous prediction models have also sought to incorporate other individual genetic variants[4]. The AD PRS summarizes many genetic variants into a single metric and has been associated with cognitive measures in previous studies among Hispanics/Latinos[5].
Lifestyle	Base + Sleep duration, Mediterranean diet score, Exercise score	Lifestyle factors such as such as diet [6–9], sleep duration [10–13] and physical activity [14–18], have all been strongly associated with cognitive function in previous studies.
Chronic Conditions	Base + Diabetes, Hypertension	Hypertension [19–22] and type 2 diabetes [23–27] have both been strongly associated with cognitive function in previous studies.
Metabolites	base + Gamma-CEHC glucuronide, 5'-Methylthioadenosine, Glucose, Mannose, Ribitol, Mannitol/sorbitol	Research from HCHS/SOL, ARIC and the Boston Puerto Rican Health Study (BPRHS) identified six metabolites consistently associated with lower cognitive function in Hispanics/Latinos and across populations[28,29]. Metabolites further reflect the role diet and type 2 diabetes among other factors in cognitive aging, and are especially useful when such information is not collected directly in studies.
Full	Base + Genetic Cognitive + Lifestyle + Metabolites + Chronic Conditions	The full model combined all domains of risk factors for cognitive measures to evaluate the totality of evidence.

Supplemental Figure 1. SHAP value plot for predicting MCI using logistic regression based on the metabolite model including diabetes (Base + diabetes, gamma-CEHC glucuronide, 5'-Methylthioadenosine, glucose, mannose, ribitol, and mannitol/sorbitol)

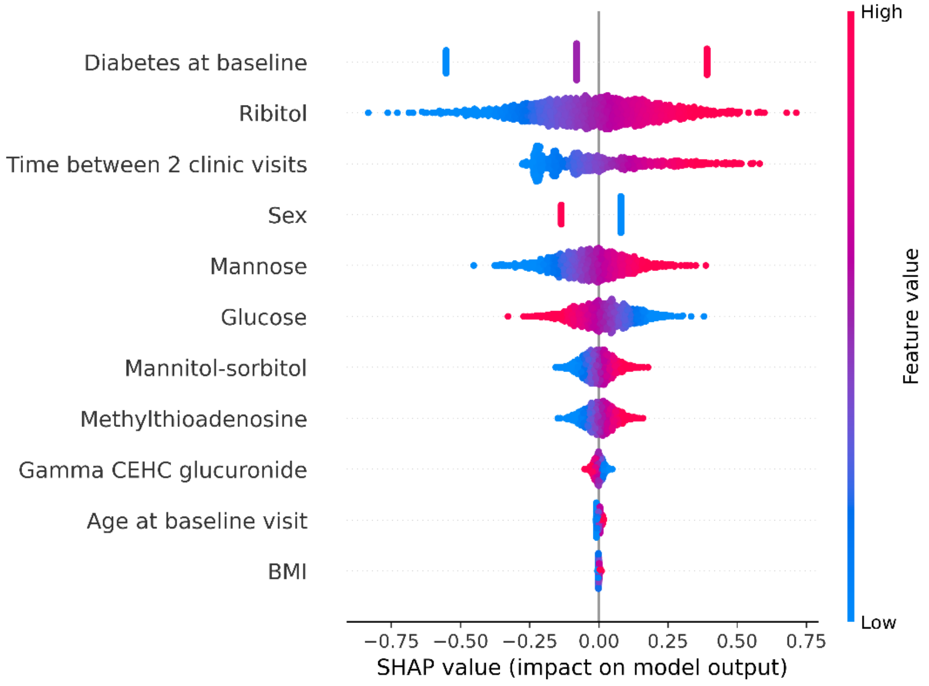

Features are listed in order of importance, from highest at the top of the plot to lowest at the bottom. Pink indicates high feature values and blue low feature values. SHAP values on the left indicate that the relevant feature contributed to lowering prediction of MCI and on the right contributed to elevating prediction of MCI. Sex male indicated by pink and sex female by blue. Having diabetes indicated by pink, pre-diabetes by purple, and not having diabetes by blue.

Supplemental Figure 2. Principal Component Analysis of Metabolites in Batch 1 vs Batch 2

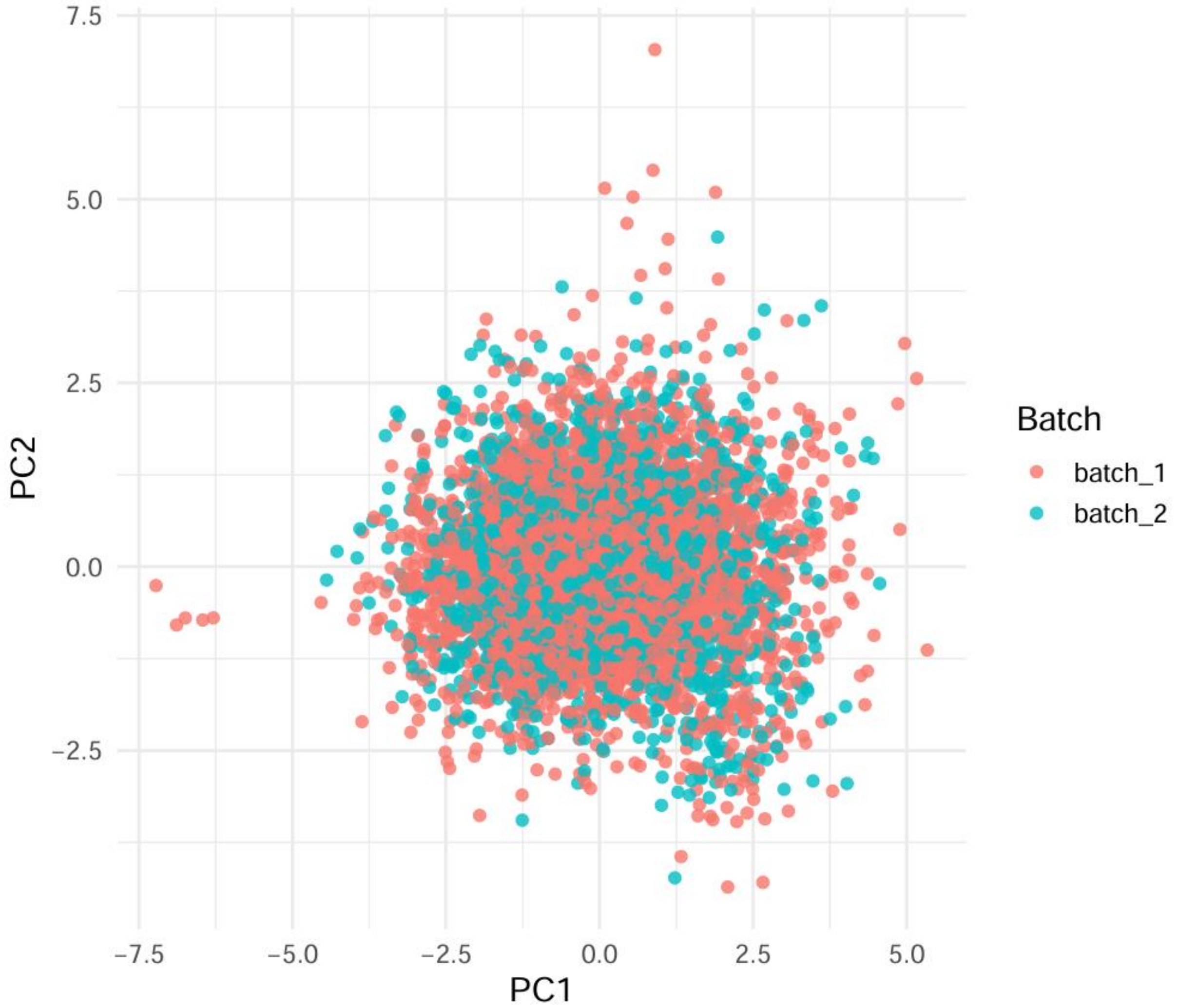

Metabolites were quantified in fasting serum using the discovery HD4 platform (Metabolon Inc Durham NC) in two "batches". The first batch (red), included 4,002 individuals sampled at random from HCHS/SOL individuals who had whole-genome sequencing data in 2017. The second batch (blue), included 2,330 participants (50 individuals overlapped with batch 1) selected based on criteria specific to HCHS/SOL sub-studies (overrepresentation of older individuals and persons with reduced estimated glomerular filtration rate) in 2021. Six metabolites were considered in prediction models: gamma-CEHC glucuronide, 5'-Methylthioadenosine, glucose, mannose, ribitol, and mannitol/sorbitol.

References

1. Hou, X.-H. *et al.* Models for predicting risk of dementia: a systematic review. *J. Neurol. Neurosurg. Psychiatr.* **90**, 373–379 (2019).
2. Tang, M. X. *et al.* The APOE-epsilon4 allele and the risk of Alzheimer disease among African Americans, whites, and Hispanics. *JAMA* **279**, 751–755 (1998).
3. Blue, E. E., Horimoto, A. R. V. R., Mukherjee, S., Wijsman, E. M. & Thornton, T. A. Local ancestry at APOE modifies Alzheimer's disease risk in Caribbean Hispanics. *Alzheimers Dement* **15**, 1524–1532 (2019).
4. Rowe, T. W. *et al.* Machine learning for the life-time risk prediction of Alzheimer's disease: a systematic review. *Brain Commun.* **3**, fcab246 (2021).
5. Sofer, T. *et al.* A polygenic risk score for Alzheimer's disease constructed using APOE-region variants has stronger association than APOE alleles with mild cognitive impairment in Hispanic/Latino adults in the U.S. *Alzheimers Res Ther* **15**, 146 (2023).
6. Moustafa, B. *et al.* Association of mediterranean diet with cognitive decline among diverse hispanic or latino adults from the hispanic community health study/study of latinos. *JAMA Netw. Open* **5**, e2221982 (2022).
7. Sapkota, S. *et al.* Multimodal Associations of Modifiable Risk Factors on White Matter Injury: The SOL-INCA-MRI Study (HCHS/SOL). *Stroke* (2025) doi:10.1161/STROKEAHA.124.049904.
8. Mattei, J. *et al.* The Mediterranean Diet and 2-Year Change in Cognitive Function by Status of Type 2 Diabetes and Glycemic Control. *Diabetes Care* **42**, 1372–1379 (2019).
9. Boumenna, T. *et al.* MIND diet and cognitive function in puerto rican older adults. *J. Gerontol. A Biol. Sci. Med. Sci.* **77**, 605–613 (2022).
10. Ramos, A. R. *et al.* Sleep duration and neurocognitive function in the hispanic community health study/study of latinos. *Sleep* **39**, 1843–1851 (2016).
11. Ramos, A. R. *et al.* Sleep and neurocognitive decline in the Hispanic Community Health Study/Study of Latinos. *Alzheimers Dement* **16**, 305–315 (2020).
12. Ramos, A. R. *et al.* Association between sleep duration and the mini-mental score: the Northern Manhattan study. *J. Clin. Sleep Med.* **9**, 669–673 (2013).
13. Agudelo, C. *et al.* Actigraphic sleep patterns and cognitive decline in the Hispanic Community Health Study/Study of Latinos. *Alzheimers Dement* **17**, 959–968 (2021).
14. Vásquez, E. *et al.* Is there a relationship between accelerometer-assessed physical activity and sedentary behavior and cognitive function in US Hispanic/Latino adults? The Hispanic Community Health Study/Study of Latinos (HCHS/SOL). *Prev. Med.* **103**, 43–48 (2017).
15. Piedra, L. M. *et al.* The influence of exercise on cognitive function in older hispanic/latino adults: results from the "¡caminemos!" study. *Gerontologist* **57**, 1072–1083 (2017).
16. Wilbur, J. *et al.* The relationship between physical activity and cognition in older Latinos. *J. Gerontol. B Psychol. Sci. Soc. Sci.* **67**, 525–534 (2012).
17. Halloway, S., Wilbur, J., Schoeny, M. E. & Barnes, L. L. The relation between physical activity and cognitive change in older latinos. *Biol. Res. Nurs.* **19**, 538–548 (2017).

18. Shih, I.-F., Paul, K., Haan, M., Yu, Y. & Ritz, B. Physical activity modifies the influence of apolipoprotein E ε4 allele and type 2 diabetes on dementia and cognitive impairment among older Mexican Americans. *Alzheimers Dement* **14**, 1–9 (2018).
19. Tarraf, W. *et al.* Blood pressure and hispanic/latino cognitive function: hispanic community health study/study of latinos results. *J Alzheimers Dis* **59**, 31–42 (2017).
20. Márquez, F. *et al.* Hypertension, Cognitive Decline, and Mild Cognitive Impairment Among Diverse Hispanics/Latinos: Study of Latinos-Investigation of Neurocognitive Aging Results (SOL-INCA). *J Alzheimers Dis* **97**, 1449–1461 (2024).
21. de Havenon, A. *et al.* Hispanic ethnicity and risk of incident cognitive impairment in relation to systolic blood pressure. *Hypertension* **78**, 1665–1666 (2021).
22. Pacholko, A. & Iadecola, C. Hypertension, neurodegeneration, and cognitive decline. *Hypertension* **81**, 991–1007 (2024).
23. González, H. M. *et al.* Diabetes, Cognitive Decline, and Mild Cognitive Impairment Among Diverse Hispanics/Latinos: Study of Latinos-Investigation of Neurocognitive Aging Results (HCHS/SOL). *Diabetes Care* **43**, 1111–1117 (2020).
24. Elfassy, T. *et al.* Relation of diabetes to cognitive function in hispanics/latinos of diverse backgrounds in the united states. *J. Aging Health* **31**, 1155–1171 (2019).
25. Luchsinger, J. A., Cabral, R., Eimicke, J. P., Manly, J. J. & Teresi, J. Glycemia, Diabetes Status, and Cognition in Hispanic Adults Aged 55-64 Years. *Psychosom. Med.* **77**, 653–663 (2015).
26. Wu, J. H. *et al.* Impact of diabetes on cognitive function among older Latinos: a population-based cohort study. *J. Clin. Epidemiol.* **56**, 686–693 (2003).
27. Xue, M. *et al.* Diabetes mellitus and risks of cognitive impairment and dementia: A systematic review and meta-analysis of 144 prospective studies. *Ageing Res. Rev.* **55**, 100944 (2019).
28. Granot-Hershkovitz, E. *et al.* Plasma metabolites associated with cognitive function across race/ethnicities affirming the importance of healthy nutrition. *Alzheimers Dement* **19**, 1331–1342 (2023).
29. Palacios, N. *et al.* Circulating plasma metabolites and cognitive function in a puerto rican cohort. *J Alzheimers Dis* **76**, 1267–1280 (2020).
